# Association Between Vitamin D and Musculoskeletal Injuries: A Systematic Review

**DOI:** 10.7759/cureus.82495

**Published:** 2025-04-18

**Authors:** Nader Maai, Florian A Frank, Arthur Meuris, Nando Ferreira

**Affiliations:** 1 Department of Orthopaedics and Traumatology, University Hospital Basel, Basel, CHE; 2 Center for Musculoskeletal Infections (ZMSI), University Hospital Basel, Basel, CHE; 3 Orthopaedics and Traumatology, KU Leuven, Leuven, BEL; 4 Advanced Orthopaedic Training Centre (AOTC), Stellenbosch University, Cape Town, ZAF

**Keywords:** athletic performance, muscle function, musculoskeletal injuries, stress fractures, supplementation, vitamin d

## Abstract

It is generally accepted that the maintenance of the health of the musculoskeletal system is highly dependent on vitamin D. However, the relationship between vitamin D levels and the incidence of musculoskeletal injuries is still uncertain. This systematic review aimed to summarize the available information on the association between vitamin D levels and musculoskeletal injuries. Seven electronic databases were searched using Boolean operators to link MeSH (Medical Subject Headings) terms and free text phrases. This improved the sensitivity and specificity of the search results. Studies investigating the association between vitamin D status and musculoskeletal damage were included. Twelve studies met the inclusion criteria. The majority of studies reported an increased prevalence amongst individuals with low vitamin D levels and musculoskeletal symptoms or injury. This underlines the important role of vitamin D screening and supplementation. Some studies have associated certain vitamin D metabolites with injury occurrence, suggesting that levels and balance of these metabolites may influence injury risk. However, there have been conflicting results on the effect of vitamin D supplementation on muscle function and exercise-induced muscle injury, with some studies failing to find any significant changes. Consistent evidence has demonstrated the benefits of vitamin D supplementation in reducing the incidence of stress fractures in athletes. One study suggests that genetic variations may influence how vitamin D is related to musculoskeletal health. The reviewed studies revealed a complex relationship between the vitamin D status and musculoskeletal injuries. While low vitamin D levels were consistently observed, the effects of supplementation on various musculoskeletal outcomes varied. These findings emphasize the need for further research to better understand the underlying mechanisms and to develop targeted interventions for specific populations, considering factors such as vitamin D metabolites, supplementation dosage, and genetic variations.

## Introduction and background

Vitamin D is necessary for maintaining a balance between calcium and phosphate, which is important for bone health [1]. Beyond its well-known role in bone metabolism, new research suggests that vitamin D might also be important for many other processes, including muscle function and regeneration [2]. Vitamin D insufficiency is now widely recognised as a major global public health problem [2-3]. It is estimated that over one billion people worldwide do not get enough of this essential nutrient. This has recently led to increased interest in the possible relationship between vitamin D levels and musculoskeletal injury incidence [3-4].

Musculoskeletal injuries encompass a wide range of conditions affecting bones, muscles, tendons, and ligaments, significantly impacting both individuals and healthcare systems [5-6]. These injuries can result from chronic overuse or acute traumatic events, leading to pain, disability, and a reduced quality of life. Athletes are particularly vulnerable to these issues due to the high physical demands and repetitive nature of their training and competition [7]. As a result, identifying modifiable risk factors and developing effective preventive strategies are essential in sports medicine and rehabilitation [8].

The complex relationship between vitamin D and the health of the musculoskeletal system has been the subject of much research. Vitamin D receptor (VDR) has been discovered in skeletal muscle fibres, indicating a direct effect of vitamin D on muscle function [9]. In addition, it has been shown that vitamin D can control the inflammatory response, which is an essential part of the recovery process after injury. Vitamin D has also been shown to reduce the risk of stress fractures and help maintain bone mineral density [10-12]. However, increasing numbers of studies on vitamin D levels and musculoskeletal injury have produced mixed and often conflicting findings [10-14].

Complicating the current body of knowledge are the significant differences in populations, study designs, vitamin D assays, and injury definitions. In addition, the complex nature of musculoskeletal injuries, with multiple contributing variables including age, gender, body composition, training loads, and biomechanical characteristics, makes it difficult to determine the precise role of vitamin D in injury risk and recovery. This systematic review aims to assess and summarise the existing literature on the relationship between vitamin D status and the incidence, severity, and recovery of musculoskeletal injuries in different populations to fill these knowledge gaps and provide a comprehensive understanding of the current state of evidence.

## Review

Methods

The Preferred Reporting Items for Systematic Reviews and Meta-Analyses (PRISMA) reporting guidelines [15] were followed in developing the PECO (population, exposure, comparison, outcome) procedure for this review. The target population was defined as people of different ages and ethnicities. The focus was on athletes and physically active people. Vitamin D status, including serum 25(OH)D concentration as well as vitamin D intake from food, was used as a measure of exposure without any limitations to the dosage. The comparison group was people with normal vitamin D status or who had no exposure to vitamin D insufficiency. The main outcomes of interest were the prevalence, severity, and recovery from musculoskeletal injuries. These included fractures, sprains, strains, and tendinopathies. The inclusion and exclusion criteria for this review are shown in Table 1.

**Table 1:** Review selection criteria

Inclusion criteria	Exclusion criteria
Studies investigating the association between vitamin D and musculoskeletal injuries	Animal studies or in vitro experiments
Observational studies (cohort, case-control, cross-sectional) and randomized controlled trials	Studies not reporting on musculoskeletal injuries as outcomes
Studies reporting serum 25(OH)D concentrations or dietary vitamin D intake	Studies not providing sufficient data for analysis (e.g., absence of effect sizes or confidence intervals)
Studies published in the English language	Commentaries, editorials, case reports, and review articles
Studies including participants of all ages and backgrounds, with a focus on athletes and physically active individuals	Studies with incomplete or unclear reporting of methods or results

Protocol for Database Searches

The seven electronic databases searched for this systematic review were Embase, Scopus, Web of Science, SPORTDiscus, ProQuest Dissertations & Theses Global, Cochrane Central Register of Controlled Trials (CENTRAL), and MEDLINE (via PubMed) till December 2024. To improve the sensitivity and specificity of the search results, the search strategy combined free-text keywords with Medical Subject Headings (MeSH) phrases using Boolean operators (Table 2).

**Table 2:** Search terms used in databases

Database	Search string
MEDLINE (via PubMed)	(("Vitamin D"[Mesh] OR "Cholecalciferol"[Mesh] OR "Ergocalciferols"[Mesh] OR "Vitamin D Deficiency"[Mesh] OR "vitamin d"[tiab] OR "25-hydroxyvitamin D"[tiab] OR "25(OH)D"[tiab] OR "calcidiol"[tiab] OR "calcitriol"[tiab]) AND ("Wounds and Injuries"[Mesh] OR "Fractures, Bone"[Mesh] OR "Soft Tissue Injuries"[Mesh] OR "Athletic Injuries"[Mesh] OR "Sprains and Strains"[Mesh] OR "Tendinopathy"[Mesh] OR "Tendon Injuries"[Mesh] OR "Ligaments/injuries"[Mesh] OR "injur*"[tiab] OR "fracture*"[tiab] OR "sprain*"[tiab] OR "strain*"[tiab] OR "tendin*"[tiab] OR "tendon*"[tiab] OR "ligament*"[tiab]) AND ("Cohort Studies"[Mesh] OR "Case-Control Studies"[Mesh] OR "Cross-Sectional Studies"[Mesh] OR "Randomized Controlled Trial"[pt] OR "cohort"[tiab] OR "prospective"[tiab] OR "retrospective"[tiab] OR "case-control"[tiab] OR "cross-sectional"[tiab] OR "randomized"[tiab] OR "randomised"[tiab] OR "RCT"[tiab]) AND "English"[lang])
Embase	('vitamin d'/exp OR 'cholecalciferol'/exp OR 'ergocalciferol'/exp OR 'vitamin d deficiency'/exp OR 'vitamin d':ti,ab OR '25-hydroxyvitamin d':ti,ab OR '25(oh)d':ti,ab OR 'calcidiol':ti,ab OR 'calcitriol':ti,ab) AND ('injury'/exp OR 'fracture'/exp OR 'soft tissue injury'/exp OR 'sport injury'/exp OR 'sprain'/exp OR 'strain'/exp OR 'tendinopathy'/exp OR 'tendon injury'/exp OR 'ligament injury'/exp OR injur*:ti,ab OR fracture*:ti,ab OR sprain*:ti,ab OR strain*:ti,ab OR tendin*:ti,ab OR tendon*:ti,ab OR ligament*:ti,ab) AND ('cohort analysis'/exp OR 'case control study'/exp OR 'cross-sectional study'/exp OR 'randomized controlled trial'/exp OR cohort:ti,ab OR prospective:ti,ab OR retrospective:ti,ab OR 'case control':ti,ab OR 'cross-sectional':ti,ab OR randomized:ti,ab OR randomised:ti,ab OR rct:ti,ab) AND [english]/lim
Scopus	TITLE-ABS-KEY(("vitamin d" OR cholecalciferol OR ergocalciferol OR "vitamin d deficiency" OR "25-hydroxyvitamin d" OR "25(OH)D" OR calcidiol OR calcitriol) AND (injur* OR fracture* OR sprain* OR strain* OR tendin* OR tendon* OR ligament*) AND (cohort OR prospective OR retrospective OR "case-control" OR "cross-sectional" OR randomized OR randomised OR rct)) AND LANGUAGE(english)
Web of Science	TS=(("vitamin d" OR cholecalciferol OR ergocalciferol OR "vitamin d deficiency" OR "25-hydroxyvitamin d" OR "25(OH)D" OR calcidiol OR calcitriol) AND (injur* OR fracture* OR sprain* OR strain* OR tendin* OR tendon* OR ligament*) AND (cohort OR prospective OR retrospective OR "case-control" OR "cross-sectional" OR randomized OR randomised OR rct)) AND LANGUAGE: (English)
SPORTDiscus	(DE "VITAMIN D" OR DE "VITAMIN D deficiency" OR TI ("vitamin d" OR cholecalciferol OR ergocalciferol OR "vitamin d deficiency" OR "25-hydroxyvitamin d" OR "25(OH)D" OR calcidiol OR calcitriol) OR AB ("vitamin d" OR cholecalciferol OR ergocalciferol OR "vitamin d deficiency" OR "25-hydroxyvitamin d" OR "25(OH)D" OR calcidiol OR calcitriol)) AND (DE "WOUNDS & injuries" OR DE "FRACTURES" OR DE "SOFT tissue injuries" OR DE "SPORTS injuries" OR DE "SPRAINS" OR DE "STRAINS" OR DE "TENDINOPATHY" OR DE "TENDON injuries" OR DE "LIGAMENTS -- Wounds & injuries" OR TI (injur* OR fracture* OR sprain* OR strain* OR tendin* OR tendon* OR ligament*) OR AB (injur* OR fracture* OR sprain* OR strain* OR tendin* OR tendon* OR ligament*)) AND (DE "COHORT analysis" OR DE "CASE-controlled studies" OR DE "CROSS-sectional method" OR DE "RANDOMIZED controlled trials" OR TI (cohort OR prospective OR retrospective OR "case-control" OR "cross-sectional" OR randomized OR randomised OR rct) OR AB (cohort OR prospective OR retrospective OR "case-control" OR "cross-sectional" OR randomized OR randomised OR rct))
Cochrane Central Register of Controlled Trials (CENTRAL)	([mh "Vitamin D"] OR [mh Cholecalciferol] OR [mh Ergocalciferols] OR [mh "Vitamin D Deficiency"] OR "vitamin d":ti,ab OR "25-hydroxyvitamin D":ti,ab OR "25(OH)D":ti,ab OR calcidiol:ti,ab OR calcitriol:ti,ab) AND ([mh "Wounds and Injuries"] OR [mh "Fractures, Bone"] OR [mh "Soft Tissue Injuries"] OR [mh "Athletic Injuries"] OR [mh "Sprains and Strains"] OR [mh Tendinopathy] OR [mh "Tendon Injuries"] OR [mh "Ligaments/injuries"] OR injur*:ti,ab OR fracture*:ti,ab OR sprain*:ti,ab OR strain*:ti,ab OR tendin*:ti,ab OR tendon*:ti,ab OR ligament*:ti,ab)
ProQuest Dissertations & Theses Global	noft(("vitamin d" OR cholecalciferol OR ergocalciferol OR "vitamin d deficiency" OR "25-hydroxyvitamin d" OR "25(OH)D" OR calcidiol OR calcitriol) AND (injur* OR fracture* OR sprain* OR strain* OR tendin* OR tendon* OR ligament*) AND (cohort OR prospective OR retrospective OR "case-control" OR "cross-sectional" OR randomized OR randomised OR rct)) AND la.exact("English")

Data Extraction Method

To ensure a comprehensive and trustworthy collection of data from the included studies, the data extraction technique for this systematic review was developed. Two impartial reviewers who were not part of the author team developed and tested a standard data extraction form for this review. The form was then improved based on the feedback and understanding from the pilot phase, and the final data extraction form was developed. The final data extraction form included information on study authorship, year of publication, design, country, sample size, age, gender, level of competition, sport or physical activity, exposure details (serum 25(OH)D levels, vitamin D intake, method of assessment), outcomes (type of musculoskeletal injury, incidence, severity, recovery time), and key findings (effect size, confidence limits, p-value).

Bias Assessment Protocol

Three different tools were used to assess the bias for the different methods considered in this review. For cross-sectional studies, the AXIS tool [16] was used. RCTs were assessed using the Cochrane RoB 2.0 tool [17]. To assess bias in non-randomised and observational articles, the ROBINS-I tool [18] was used.

Results

Schematic of Article Selection

**Figure 1:**
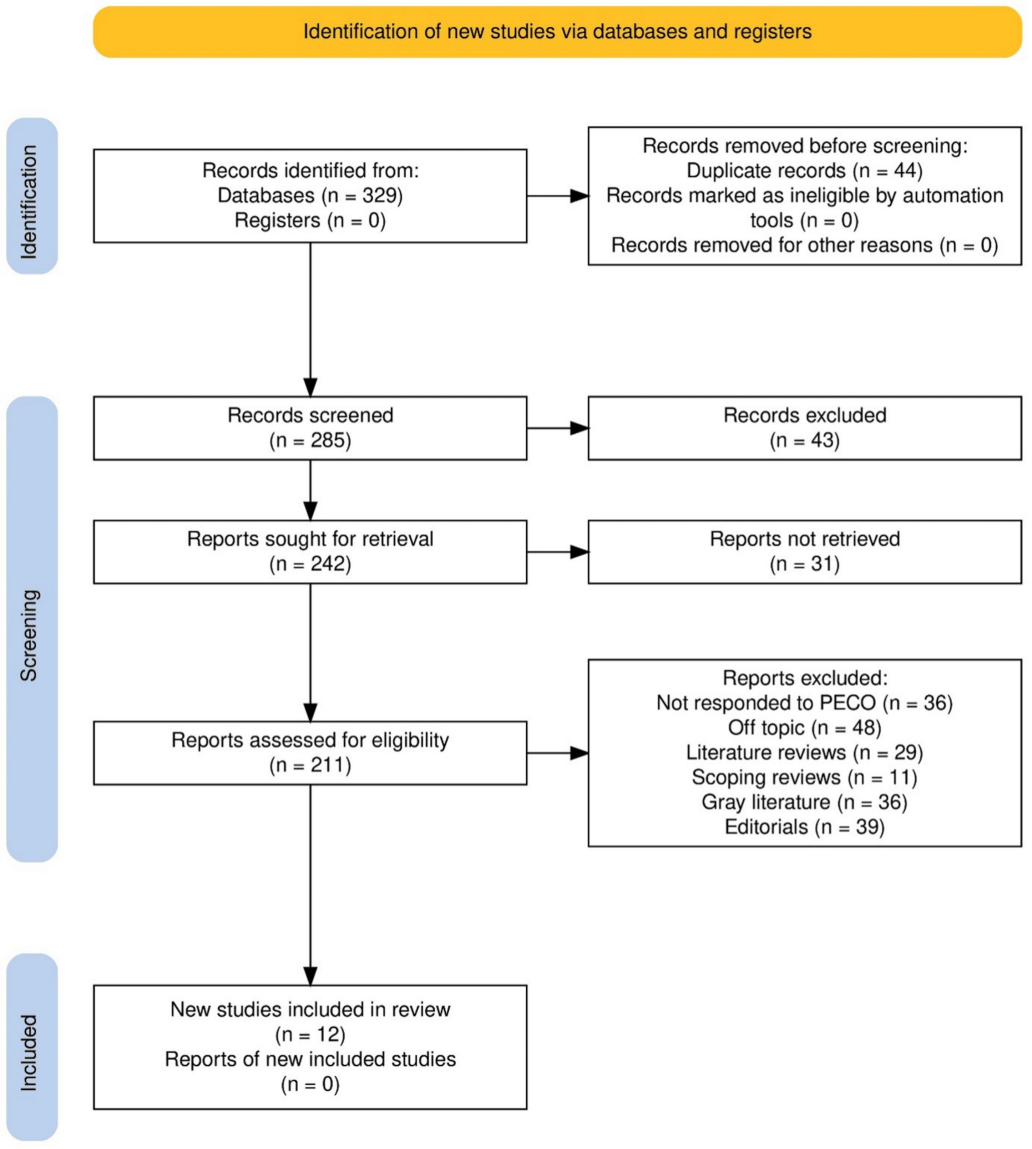
Preferred Reporting Items for Systematic Reviews and Meta-Analyses (PRISMA) protocol representation of the study inclusion process for the review

A total of 329 articles (Figure 1) were identified after a thorough search of relevant databases. No additional records were found after a manual search of all registries. Following careful screening, 285 unique records remained for further analysis, with 44 duplicates identified and eliminated from the data collected. After the elimination of 43 records due to the absence of full-text articles, 242 reports were left that needed to be retrieved. Despite extensive attempts, 31 of the 211 reports could not be retrieved due to paywall restrictions. Their eligibility was determined based on pre-specified inclusion criteria. During the eligibility assessment, 160 reports were rejected for various reasons: 36 were considered as off-topic, 48 as insufficiently PECO-compliant, 29 as literature reviews, 11 as scoping reviews, 36 as grey literature, and 40 as editorials. In the end, 12 papers [19-29] met the inclusion criteria. They were included in the final assessment.

Assessment of Bias in Different Domains

**Figure 2:**
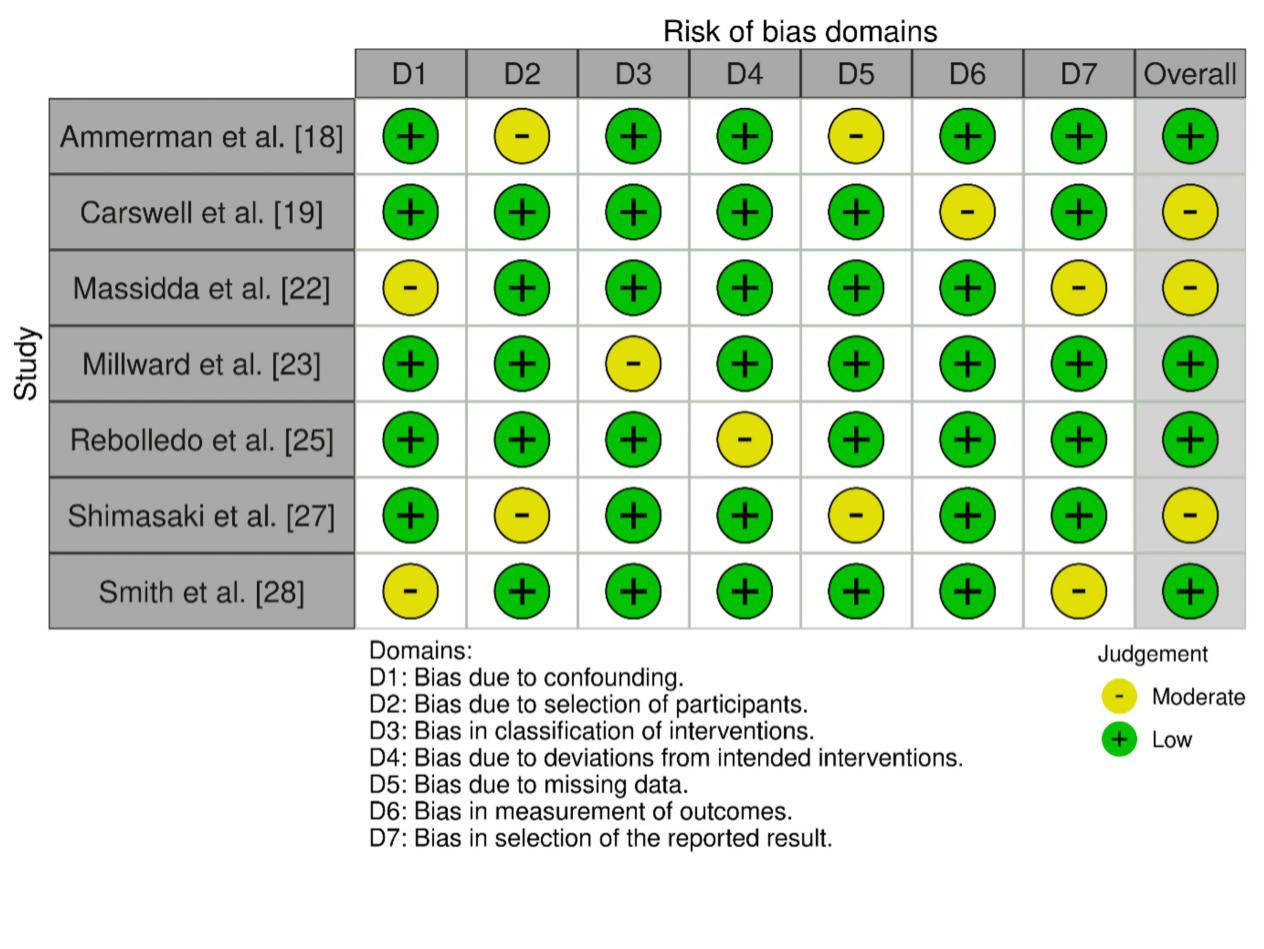
Bias assessment using the ROBINS-I tool

In the ROBINS-I assessment, three studies [18,23,25] had an overall low risk of bias (Figure 2), whereas four studies [19,22,27,28] had a moderate risk of bias. 

**Figure 3:**
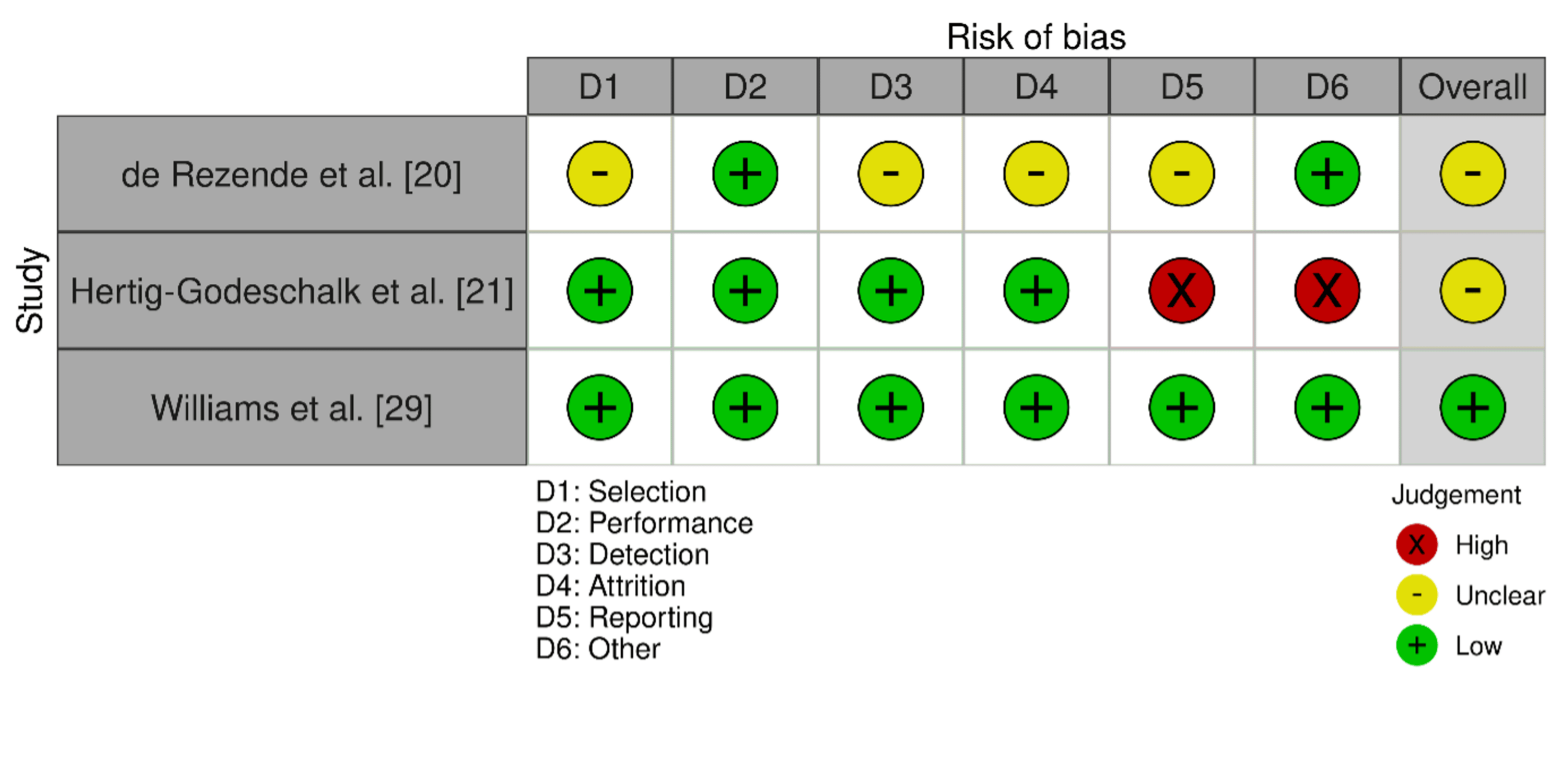
Bias assessment using the AXIS tool

The AXIS tool assessment (Figure 3) showed that one study [29] had an overall low risk of bias. 

**Figure 4:**
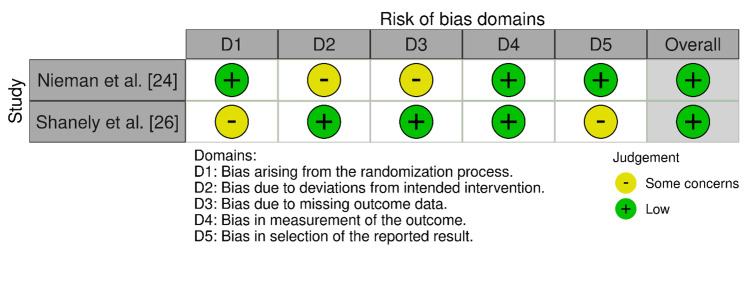
Bias assessment using the RoB 2.0 tool

Two other studies [20,21] had a moderate risk. Both studies [24,26] had an overall low risk of bias based on the RoB 2.0 tool (Figure 4), despite notable problems in specific areas.

Assessment of Demographic Variables

Table 3 shows which studies were included and analysed: RCTs [24,26], cross-sectional studies [20,21], prospective [18,19,29], and retrospective cohort studies [23] and case-control studies [27,28]. Among the included studies, there were differences in the size of the sample and the research design. The study [24] included 28 participants in a randomised, double-blind, placebo-controlled design. The participants were NASCAR pit crew athletes. The mean age of the 1,637 males and 530 females in a prospective cohort study was 22.6 ± 7.5 years [19]. Several studies have looked at specific sports groups, including NFL combine attendees [25], NCAA Division I athletes [23,29], and professional football players [22]. Other studies have included patients with acute spinal cord injury [21], high school athletes with low vitamin D status [26], female patients aged 16-40 years [18], preprofessional dancers aged 17 ± 4.44 years [20], and patients with foot or ankle injuries [28]. One study used a cumulative case-control approach to compare athletes with and without stress fractures of the fifth metatarsal [27]. 

**Table 3:** Studies that were included in the review and the assessments that were observed

Author ID	Study design and sample size	Interventions assessed	Study duration	Primary outcomes evaluated	Results observed	Overall inference drawn
Ammerman et al. [18]	Prospective cohort study; 105 female patients aged 16-40 years	Vitamin D levels (normal: ≥32 ng/mL, insufficient: 20.01-31.9 ng/mL, deficient: ≤20 ng/mL)	Within four weeks of diagnosis	Prevalence of low vitamin D in acute and overuse injuries	65.7% had low vitamin D; the highest prevalence was in ligamentous/cartilaginous injuries (76.5%)	High prevalence of low vitamin D in female patients with various musculoskeletal complaints
Carswell et al. [19]	Prospective cohort study; 1637 men and 530 women aged 22.6 ± 7.5 years	Serum 25(OH)D, 24,25(OH)2D, and 1,25(OH)2D levels	12 weeks	Incidence of lower body overuse musculoskeletal and bone stress injuries	Lower incidence associated with higher 24,25(OH)2D and lower 1,25(OH)2D:24,25(OH)2D ratio	Greater conversion of 25(OH)D to 24,25(OH)2D relative to 1,25(OH)2D associated with lower injury incidence
de Rezende et al. [20]	Cross-sectional study; 49 pre-professional dancers aged 17 ± 4.44 years	Serum 25(OH)D3 levels (normal: >30 ng/mL, insufficient/deficient: <30 ng/mL)	Six months	Muscle function (peak torque, fatigue) and injury incidence	The normal 25(OH)D3 group had lower fatigue rates; injured dancers had lower peak torque	Link between serum 25(OH)D3 and reduced muscle fatigue resistance; association between muscular strength and injury
Hertig-Godeschalk et al. [21]	Cross-sectional study; 87 patients with acute spinal cord injury (SCI)	Vitamin D status (deficient: ≤50 nmol/L, insufficient: 50-75 nmol/L, sufficient: >75 nmol/L)	Median 15 days after the SCI onset	Prevalence of deficient, insufficient, and sufficient vitamin D status	67% deficient, 25% insufficient; negative correlation with BMI, positive correlation with calcium status	High prevalence of deficient or insufficient vitamin D status directly after SCI onset; recommendation for supplementation
Massidda et al. [22]	Cohort study; 54 male professional football players	VDR gene polymorphisms (ApaI, BsmI, FokI)	Four football seasons (2009-2013)	Association between VDR polymorphisms and musculoskeletal injury (MI) incidence and severity	No significant differences in MI incidence/severity between genotypes; ApaI genotypes accounted for 18% of injury severity variance	ApaI genotypes may influence the severity of muscle injury in top-level football players
Millward et al. [23]	Cohort study; 802 NCAA Division I athletes (497 men, 305 women)	Vitamin D supplementation for athletes with levels <40 ng/mL	2012-2018	Stress fracture rate differences between those who improved or maintained vitamin D levels ≥40 ng/mL and those who did not	12% higher stress fracture rate in those who remained low in vitamin D compared to those who improved levels to ≥40 ng/mL; higher baseline vitamin D in outdoor vs. indoor athletes	Correcting low serum vitamin D levels reduces stress fracture risk; indoor athletes may be at greater risk for vitamin D deficiency
Nieman et al. [24]	Randomized, double-blind, placebo-controlled study; 28 NASCAR pit crew athletes	Six-week vitamin D2 supplementation (3800 IU/day) vs. placebo	6 weeks	Influence on muscle function, eccentric exercise-induced muscle damage (EIMD), and delayed onset of muscle soreness (DOMS)	Increased serum 25(OH)D2, decreased 25(OH)D3; no effect on muscle function; higher muscle damage biomarkers post-eccentric exercise in vitamin D2 group	Six-week vitamin D2 supplementation did not improve muscle function and amplified muscle damage markers following eccentric exercise
Rebolledo et al. [25]	Retrospective case series; 214 NFL combine athletes	Serum 25-hydroxyvitamin D levels (normal: ≥32 ng/mL, insufficient: 20-31 ng/mL, deficient: <20 ng/mL)	Not specified	Association between vitamin D levels and prevalence of lower extremity muscle strains and core muscle injuries	Inadequate vitamin D in 59%; lower vitamin D levels associated with muscle strain/core muscle injury history; higher odds of hamstring injury with inadequate vitamin D	High prevalence of inadequate vitamin D in NFL combine athletes; players with a muscle strain/core muscle injury history had lower vitamin D levels
Shanely et al. [26]	Randomized controlled trial; high school athletes with low vitamin D status	Portobello mushroom powder (600 IU/d vitamin D2) vs. placebo	Six weeks	Effect on skeletal muscle function and exercise-induced muscle damage	Increased serum 25(OH)D2, decreased 25(OH)D3; no differences in muscle function or muscle damage markers between groups	600 IU/d vitamin D2 did not improve muscular function or attenuate exercise-induced muscle damage in high school athletes
Shimasaki et al. [27]	Cumulative case-control study; 37 athletes (18 with 5-MT stress fractures, 19 controls)	Serum 25-hydroxyvitamin D (25-OHD), parathyroid hormone (PTH), and bone turnover markers	Not specified	Endocrine risk factors for fifth metatarsal (5-MT) stress fractures	Insufficient 25-OHD levels (<30 ng/mL), higher PTH, and higher bone-specific alkaline phosphatase were associated with increased odds of 5-MT stress fractures	25-OHD insufficiency was associated with an increased incidence of 5-MT stress fractures, which may guide interventions for prevention
Smith et al. [28]	Prospective case-control study; 75 patients with foot or ankle injuries	Serum 25-OH vitamin D levels in patients with low-energy fractures vs. ankle sprains	Six months	Prevalence of vitamin D deficiency in patients with foot or ankle disorders	47% had insufficient vitamin D (<30 ng/mL), 13% deficient (<20 ng/mL); lower vitamin D in fracture patients vs. ankle sprains; smoking, obesity, and medical risk factors associated with insufficiency	Hypovitaminosis D was common in patients with foot or ankle injuries, particularly in those with low-energy fractures and certain risk factors
Williams et al. [29]	Prospective cross-sectional analysis with retrospective control comparison; 118 NCAA Division I athletes	Vitamin D3 supplementation (50,000 IU/week for eight weeks) in athletes with serum 25(OH)D <30 ng/mL	Eight weeks	Effect of vitamin D3 supplementation on stress fracture occurrence in high-risk collegiate athletes	Almost half of the athletes were vitamin D deficient; stress fracture rate decreased from 7.51% (retrospective) to 1.65% (prospective) with supplementation	Vitamin D3 supplementation significantly reduced stress fracture incidence in high-risk collegiate athletes

Evaluation of Parameters and Outcomes

Ammerman et al. [18] investigated the incidence of low vitamin D levels in patients diagnosed with acute and overuse injuries within four weeks. The relationship between blood levels of different types of vitamin D and the likelihood of stress fractures and overuse injuries in the lower body over 12 weeks was investigated by Carswell et al. [19]. The study by De Rezende et al. [20] looked at the relationship between vitamin D levels in the blood, injuries, and muscle function over six months. 

Approximately 15 days after injury, Hertig-Godeschalk and colleagues [21] measured the proportion of participants with low, inadequate, or adequate vitamin D. Massidda and colleagues [22] examined the relationship between variations in genes linked to vitamin D and the frequency and severity of injuries sustained by football players over four seasons. Millward et al. [23] compared athletes who maintained or increased their vitamin D levels with those who did not, to determine the number of stress fractures that occurred after treatment of individuals with low vitamin D levels. The research project was carried out from 2012 to 2018. Nieman et al. [24] investigated the effects of 3800 IU/day of vitamin D2 supplementation for six weeks on muscle performance, muscle injury during prolonged exercise, and delayed muscle soreness in comparison with a placebo. Although they did not specify the duration of the study, Rebolledo et al. [25] investigated the relationship between blood levels of vitamin D and the frequency of leg and trunk muscle strains. Shanely et al. [26] investigated the effects of vitamin D2 (600 IU/d) and mushroom powder on exercise-induced muscle damage and function over six weeks. Shimasaki et al. [27] examined hormonal risk variables for stress fractures in a predetermined foot bone. These included vitamin D, parathyroid hormone, and blood-borne turnover markers, although they did not specify a period. Over a period of six months, Smith et al. [28] investigated the prevalence of vitamin D deficiency in individuals with low-impact fractures compared with those with ankle sprains. The effect of vitamin D supplementation (50,000 IU/week for eight weeks) on the incidence of stress fractures in high-risk college athletes with low vitamin D levels was investigated by Williams et al. [29].

Observed Outcomes

According to a study by Ammerman et al. [18], 65.7% of the population had insufficient levels of vitamin D, with the highest percentage (76.5%) being among those who had suffered cartilage or ligament damage. Carswell et al. [19] found that when specific forms of vitamin D were balanced in the body, there were fewer bone stress injuries and lower body overuse injuries. Research by De Rezende et al. [20] showed that dancers with normal vitamin D levels had less fatigue, but those with injuries had weaker muscles. Sixty-seven percent of people had low vitamin D, which was associated with lower BMI and higher calcium levels, according to Hertig-Godeschalk et al. [21]. Massidda et al. [22] found that specific genetic variants, not frequencies, accounted for 18% of the variation in injury severity among athletes. Despite an increase in their vitamin D levels, Millward et al. [23] observed that athletes who played in outdoor sports had a 12% higher risk of stress fractures than those who played in indoor sports. Although vitamin D2 supplementation altered vitamin D levels, it did not affect muscle function, according to Nieman et al. [24]. In addition, the supplement group had higher levels of indicators of muscle damage after a period of vigorous exercise. Rebolledo et al. [25] reported that 59% of participants had vitamin D insufficiency, which was associated with increased injury risk and hamstring and trunk injuries. According to Shanely et al. [26], supplements altered vitamin D levels but did not affect muscle function or injury indicators. A specific stress fracture of the foot bone was found to be more common in people with low levels of vitamin D, high levels of bone-specific alkaline phosphatase, and high levels of parathyroid hormone, according to Shimasaki et al. [27]. Similarly, Smith et al. [28] found that 13% of participants had a vitamin D deficiency (<20 ng/mL), while 47% had insufficient levels (<30 ng/mL). Both deficiencies were linked to health risks, including being overweight and smoking. In comparison with people with ankle sprains, people with ankle fractures had lower levels. The use of supplements by athletes reduced their risk of stress fractures from 7.51% to 1.65%. Williams et al. [29] found that almost 50% of athletes had inadequate vitamin D levels.

Discussion

The relationship between vitamin D and musculoskeletal health is demonstrated by the fact that the results of the included studies differ from each other. The majority of the included papers, including those by Ammerman et al. [18], Hertig-Godeschalk et al. [21], Rebolledo et al. [25], and Smith et al. [28], consistently showed a significant incidence of low vitamin D levels in their respective populations. These studies highlight the importance of assessing and supplementing these populations with vitamin D. This suggests that people with musculoskeletal conditions or injuries often have low levels of vitamin D. 

Vitamin D is essential for the maintenance of strong, healthy bones. Because vitamin D facilitates calcium absorption from food, low levels can cause a small increase in a calcium-regulating hormone, accelerating changes in bone structure [30]. Low levels of vitamin D [30-32] and high levels of this calcium-regulating hormone [31] are linked to stress breakage, but taking supplements of both vitamins can reduce stress breakage by up to 20% [30].

Research suggests that vitamin D may play an important role in reducing acute muscle damage caused by intense physical activity [33-40]. One study [33] found that having higher levels of vitamin D before exercising was associated with muscles regaining strength more quickly after exercise-induced muscular damage. Although these experiments were not carried out in athletes in particular, another study [34] has shown that vitamin D supplementation can significantly reduce the muscle damage that occurs after high-intensity, long-duration exercise.

In our review of the current literature in this area, we found that a large number of studies have consistently shown a significant prevalence of vitamin D insufficiency or deficiency in individuals with muscle and bone complaints or problems [18,21,25,28]. This is concordant with studies by Sikora-Klak et al. [41] and Yoon et al. [42] who also reported that athletes, particularly those who compete indoors and during the winter, have a significant prevalence of vitamin D deficiency. According to Yoon et al. [42], low vitamin D levels weaken muscles and increase the risk of bone and muscle injuries. According to Sikora-Klak et al. [41], athletes who do not get enough vitamin D may be more susceptible to illness, more prone to stress fractures, and slower to recover from muscle injuries.

There may be an association between vitamin D levels and injury frequency, according to our findings [19,27]. The studies by Sikora-Klak et al. [41], Yoon et al. [42], Chevalley et al. [43], and LeBoff et al. [44] do not discuss this conclusion in detail. Nevertheless, these findings underscore the importance of monitoring blood vitamin D levels in both the general population and in athletes.

This review noted inconsistent effects of vitamin D supplementation on muscle function and exercise-induced muscle injury [20,24,26]. This inconsistency is not specifically addressed in the research by Sikora-Klak et al. [41], Yoon et al. [42], Chevalley et al. [43], and LeBoff et al. [44]. Yoon et al. [42] suggested vitamin D supplementation with 2000-6000 IU of vitamin D3 daily. This was a recommendation for athletes whose vitamin D levels were low.

There is consistent data to suggest that athletes who take vitamin D supplements have a lower risk of stress fractures, which is in line with our findings [23,29]. This finding is consistent with the recommendations of Sikora-Klak and colleagues [41] and Yoon and colleagues [42] for the treatment of athletes with low vitamin D levels to reduce their risk of injury. Vitamin D supplementation showed no beneficial effects on bone and muscle health in older people with normal blood levels of the vitamin, according to the study by LeBoff et al. [44]. In addition, hereditary variables may influence the interaction between vitamin D and muscle and bone health, as noted in our review [22]. The studies by Sikora-Klak et al. [41], Yoon et al. [42], Chevalley et al. [43], and LeBoff et al. [44] did not investigate this aspect. Although the studies by Sikora-Klak et al. [41], Yoon et al. [42], Chevalley et al. [43], and LeBoff et al. [44] did not directly evaluate the primary outcomes analyzed in this review, they were included in the discussion to provide contextual relevance and to support broader clinical implications regarding vitamin D and musculoskeletal health. These studies contributed valuable insights into vitamin D recommendations, population-specific supplementation thresholds, and its potential preventive role in injury mitigation, particularly among athletes and active individuals. Sikora-Klak et al. [41] and Yoon et al. [42] advocated for targeted supplementation strategies in athletic populations, which paralleled the associations observed in our analysis regarding reduced stress fracture risk. Although Chevalley et al. [43] and LeBoff et al. [44] did not focus on athletic cohorts or injury incidence per se, their observations on the variability of musculoskeletal outcomes with respect to age, baseline vitamin D status, and genetic predispositions provided supporting evidence that helped explain some of the inconsistencies identified in the pooled studies.

Limitations

The main limitation of our review was the diversity of the included studies, which were very different in terms of the characteristics of the population, the techniques used to assess vitamin D, and the musculoskeletal outcomes that they investigated. It was difficult to perform a meta-analysis and draw definitive conclusions because of this heterogeneity. The quality of the methods used in the included studies was also variable, and the validity of their results was affected by small sample sizes and the lack of appropriate controls. Furthermore, as most of the studies were observational, it was difficult to establish a direct link between vitamin D levels and musculoskeletal problems.

Clinical Recommendations

Based on the results of this thorough analysis, several recommendations can be made to improve therapeutic practice and to increase our knowledge of the relationship between vitamin D and musculoskeletal problems. First, to establish causal relationships between vitamin D levels and musculoskeletal outcomes, large, carefully designed RCTs are needed. These trials should consider a range of variables, including vitamin D metabolites, supplement dose, and population characteristics. Second, standardised techniques for the diagnosis of musculoskeletal problems and the measurement of vitamin D levels should be used to enable comparisons between trials and meta-analyses to be carried out. In addition, the use of supplements and regular vitamin D testing should be considered for people who are more prone to musculoskeletal injuries, such as athletes and patients with specific risk factors. Further studies are needed to understand how genetic variants may affect the relationship between vitamin D and musculoskeletal health so that specific treatments and risk assessment methods can be developed.

## Conclusions

As low vitamin D levels are often associated with musculoskeletal problems or complaints, the results of our analysis highlight the need to measure and provide vitamin D supplementation in these populations. Evidence is inconsistent regarding the effect of vitamin D supplementation on various musculoskeletal outcomes, such as muscle function and exercise-induced muscle injury. The importance of vitamin D metabolites and genetic variants in controlling the relationship between vitamin D and musculoskeletal health is complex. Therefore, while an association is observed, causality is not established, and further research is needed to clarify these relationships.
